# Citrullinated proteins have increased immunogenicity and arthritogenicity and their presence in arthritic joints correlates with disease severity

**DOI:** 10.1186/ar1697

**Published:** 2005-02-21

**Authors:** Karin Lundberg, Suzanne Nijenhuis, Erik R Vossenaar, Karin Palmblad, Walter J van Venrooij, Lars Klareskog, AJW Zendman, Helena Erlandsson Harris

**Affiliations:** 1 Rheumatology Unit, Department of Medicine, Karolinska Institutet, Stockholm, Sweden; 2 Department of Biochemistry, Radboud University Nijmegen, Nijmegen, The Netherlands

## Abstract

Autoantibodies directed against citrulline-containing proteins have an impressive specificity of nearly 100% in patients with rheumatoid arthritis and have been suggested to be involved in the disease pathogenesis. The targeted epitopes are generated by a post-translational modification catalysed by the calcium-dependent enzyme peptidyl arginine deiminase (PAD), which converts positively charged arginine to polar but uncharged citrulline. The aim of this study was to explore the effects of citrullination on the immunogenicity of autoantigens as well as on potential arthritogenicity. Thus, immune responses to citrullinated rat serum albumin (Cit-RSA) and to unmodified rat serum albumin (RSA) were examined as well as arthritis development induced by immunisation with citrullinated rat collagen type II (Cit-CII) or unmodified CII. In addition, to correlate the presence of citrullinated proteins and the enzyme PAD4 with different stages of arthritis, synovial tissues obtained at different time points from rats with collagen-induced arthritis were examined immunohistochemically. Our results demonstrate that citrullination of the endogenous antigen RSA broke immunological tolerance, as was evident by the generation of antibodies directed against the modified protein and cross-reacting with the native protein. Furthermore we could demonstrate that Cit-CII induced arthritis with higher incidence and earlier onset than did the native counterpart. Finally, this study reveals that clinical signs of arthritis precede the presence of citrullinated proteins and the enzyme PAD4. As disease progressed into a more severe and chronic state, products of citrullination appeared specifically in the joints. Citrullinated proteins were detected mainly in extracellular deposits but could also be found in infiltrating cells and on the cartilage surface. PAD4 was detected in the cytoplasm of infiltrating mononuclear cells, from day 21 after immunisation and onwards. In conclusion, our data reveal the potency of citrullination to break tolerance against the self antigen RSA and to increase the arthritogenic properties of the cartilage antigen CII. We also show that citrullinated proteins and the enzyme PAD4 are not detectable in healthy joints, and that the appearance and amounts in arthritic joints of experimental animals are correlated with the severity of inflammation.

## Introduction

The chronic inflammatory joint disease rheumatoid arthritis (RA) is characterised by synovial inflammation and pannus formation, which can lead to severe destruction of cartilage and bone. Several self proteins have been suggested as disease-driving autoantigens, and the presence of autoantibodies with different specificities in patients with RA (reviewed in [1,2]) supports the hypothesis of an autoimmune aetiology. Rheumatoid factor has for a long time been the best-described RA-associated antibody marker, recognising the Fc part of IgG molecules. However, another class of autoantibodies has lately gained attention, namely antibodies directed against proteins containing the non-standard amino acid citrulline [3,4].

Citrulline is generated by the deimination of arginine, a post-translational modification occurring during apoptosis as well as during the terminal differentiation of cells, in both healthy and arthritic individuals [5,6]. Citrullination is catalysed by a family of calcium-dependent enzymes named peptidyl arginine deiminase (PAD, EC 3.5.3.15) (reviewed in [7]). These enzymes are present in several different cell and tissue types, including inflammatory cells (PAD2 [8-10] and PAD4 [10-12]). PAD4 has been detected in granulocytes infiltrating the synovial tissue in a mouse model of arthritis [13] and this enzyme, together with PAD2, has also been demonstrated in macrophages from synovial fluid of patients with RA [10].

The best-described citrulline-reactive autoantibodies associated with RA are the following: anti-perinuclear factor [14,15] and anti-keratin autoantibodies [16,17], both directed against citrullinated filaggrin [18]; anti-Sa autoantibodies [19] directed against citrullinated vimentin [20]; and antibodies against cyclic citrullinated peptide (anti-CCP) [21,22]. These latter autoantibodies have a sensitivity of up to 80% and a specificity of 98% in patients with RA [1,22]. Besides this high specificity, these markers are present early in disease, even before clinical onset [23,24], and they are synthesised locally by plasma cells in the pannus [25,26]. In addition, the existence of citrulline-reactive antibodies has been associated with a more active and severe disease [27-34] and a strong association with major histocompatibility complex (MHC) shared epitope haplotypes [28,35,36] has also been reported.

The accumulated data point towards a link between citrullinated proteins and the pathogenesis of RA. We therefore considered it to be of interest to explore the effects of citrullination on the immunogenicity of autoantigens and on potential arthritogenicity. In the present study we examined the responses of rat T and B cells to citrullinated rat serum albumin (Cit-RSA) in comparison with those of unmodified rat serum albumin (RSA). To investigate the clinical arthritogenic relevance of citrullination, the cartilage antigen rat collagen type II (CII) was modified and arthritis development was evaluated in the experimental rat model collagen-induced arthritis (CIA). In addition, to correlate the presence of citrullinated proteins with that of PAD4 with different stages of arthritis, we examined synovial tissue immunohistochemically at different time points of CIA.

Our study demonstrates, for the first time, the kinetics of the presence of citrullinated proteins as well as the enzyme PAD4 in arthritic joints from experimental animals. The amounts of citrullinated proteins and the enzyme PAD4 are correlated with severity of inflammation and are not detectable in healthy joints. The study also reveals that the citrullination of proteins can break natural tolerance mechanisms and increase the arthritogenic properties of CII.

## Materials and methods

### Animals

Lew.1AV1 and Dark Agouti (DA) rats, originating from the Zentralinstitut für Versuchstierzucht, Hannover, Germany, were bred, kept and used under pathogen-free conditions at the animal department of Karolinska University Hospital, Stockholm, Sweden. Animals were fed with standard rodent food and water *ad libitum*. Experiments were performed with female Lew.1AV1 or male DA rats, aged 9 to 14 weeks, with approval from the Stockholm North Ethical Committee.

### Preparation and citrullination of antigen

CII was prepared from Swarm's rat chondrosarcoma by pepsin digestion and purification as previously described [37,38]. CII was stored freeze-dried at -20°C until dissolved in 0.01 M acetic acid and dialysed against the citrullination buffer, 0.1 M Tris-HCl (pH 7.6) containing 10 mM CaCl_2 _and 5 mM dithiothreitol. RSA (Sigma-Aldrich, Steinheim, Germany) was dissolved directly in citrullination buffer. Proteins were incubated with rabbit skeletal muscle PAD (Sigma), at a concentration of 2 U/mg protein, for 2 hours at 37°C. Citrullination was terminated by adding 20 mM EDTA and subsequently dialysed successively against 2 mM EDTA (pH7.6) and Milli-Q water containing 10 mM Tris-HCl (pH7.6) at 4°C. Control proteins were treated similarly, apart from the addition of PAD. Protein solutions were then freeze-dried and stored at -20°C. For experimental use, freeze-dried proteins were dissolved in appropriate buffer, namely RSA in PBS and CII in 0.01 M acetic acid.

**Figure 1:**
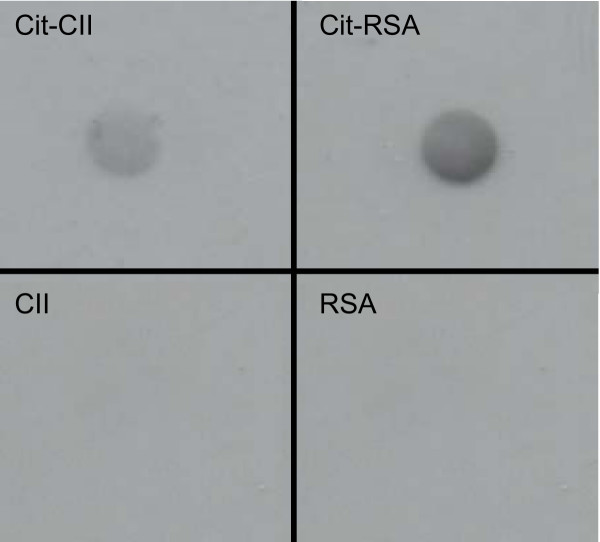
Verification of citrullination of collagen type II (CII) and rat serum albumin (RSA) by immunoblotting. Using the recombinant anti-citrullinated protein antibody RA3, citrulline was detected in the citrullinated CII (Cit-CII) sample (upper left) as well as in the citrullinated RSA (Cit-RSA) sample (upper right), while uncitrullinated CII (lower left) and uncitrullinated RSA (lower right) were negative for citrulline.

To verify the purity and amount of proteins, aliquots of samples were run in SDS-PAGE gels followed by Coomassie Brilliant Blue staining. Total protein quantification was also performed with a modified version of the Bradford method, using the Coomassie Plus Protein Assay Reagent Kit (Pierce Biotechnology, Rockford, IL, USA) in accordance with the manufacturer's instructions. To verify successful citrullination, immunoblotting was performed with RA3 [39], a human recombinant antibody directed against citrulline (Fig. 1). In brief, 5 μl of citrullinated CII (Cit-CII), CII, Cit-RSA and RSA (concentration 2 mg/ml) were applied to a nitrocellulose membrane (BioTrace NT, product no. 66485; Pall Life Sciences, Ann Arbor, MI, USA). The membrane was incubated for 1 hour in blocking buffer (PBS containing 0.05% Tween 20 with 5% (w/v) non-fat dried milk) then incubated for a further 1 hour with primary antibody RA3, diluted 1:5 in blocking buffer, at room temperature (20–25°C). After incubation with a horseradish peroxidase (HRP)-conjugated donkey anti-human antibody (diluted 1:1,000 in blocking buffer; Amersham Pharmacia Biotech, Little Chalfont, Bucks., UK) for 1 hour at room temperature, the protein–antibody complexes were detected by enhanced chemiluminescence with the ECL system (Amersham Pharmacia Biotech, Uppsala, Sweden).

### Induction and evaluation of clinical disease

To increase the likelihood of detecting citrullinated proteins and the enzyme PAD4 in the joints of arthritic animals, we chose to perform the immunohistochemical stainings in the DA rat, because this strain has proven to develop the most severe CIA.

In contrast, the Lew.1AV1 rat strain develops a milder disease and was therefore selected when investigating the additive arthritogenic effects of Cit-CII. Lew.1AV1 rats were also used when studying the effects of citrullination on tolerance to RSA.

Anaesthetised rats were immunised in the base of the tail: male DA rats with 150 μg of CII dissolved in 100 μl of 0.01 M acetic acid, emulsified with an equal volume of Freund's incomplete adjuvant (FIA; Difco, Detroit, MI, USA) (intradermally); female Lew.1AV1 rats with 120 μg of CII or Cit-CII dissolved in 100 μl of 0.01 M acetic acid plus 100 μl of FIA (intradermally) or with 180 μg of RSA or Cit-RSA dissolved in 150 μl of PBS plus 150 μl FIA (subcutaneously). The animals immunised with CII and Cit-CII were monitored for signs of arthritis from day 12 after immunisation, in accordance with a previously described procedure [40]. Each paw was divided into three groups of joints, the interphalangeal joints of the digits, the metacarpophalangeal and wrist joints in the forepaws and the metatarsophalangeal and ankle joints in the hind paws. In brief, 1 point signifies swelling of one group of joints, 2 points signifies two groups of swollen joints, 3 points signifies three groups of swollen joints and 4 points signifies swelling of the entire paw. The maximum score was thus 16 for each rat.

### Evaluation of serum anti-RSA and anti-Cit-RSA antibody levels

Individual serum samples from Lew.1AV1 rats immunised with RSA and Cit-RSA were obtained at different time points, namely 12, 24 and 35 days after immunisation (by tail bleeding) as well as at 61 days after immunisation (by heart puncture). ELISA plates (96-well Maxisorp; Nunc, Roskilde, Denmark) were coated with 10 μg/ml RSA or Cit-RSA (diluted in PBS), overnight at 4°C. Plates were washed with PBS-Tween (0.05%) and sera (serially diluted in PBS containing 0.05% Tween) were added in duplicate. After 2 hours of incubation at room temperature, plates were washed as above and incubated with alkaline phosphatase-conjugated goat anti-rat IgG (diluted 1:5,000 in PBS containing 0.05% Tween; Jackson Immunoresearch Lab, West Grove, PA, USA) for a further 2 hours at room temperature. Finally, a phosphatase substrate (Sigma, St Louis, MO, USA) was added and absorbance was determined at 405 nm with an Emax precision microplate reader (Molecular Devices).

To investigate the risk of contamination by PAD in our Cit-RSA sample, giving a false positive result in our Cit-RSA ELISA, we performed an anti-PAD ELISA. Plates were coated with the same amount of PAD expected to contaminate the Cit-RSA sample in the Cit-RSA ELISA, namely 0.0432 μg/ml. The ELISA was performed with the same procedure as described for Cit-RSA and RSA (see above).

In addition, to quantify antibodies specific for anti-citrullinated protein, an in-house CCP ELISA was used. Control reactions with corresponding cyclic arginine-containing peptides were also included. In brief, Streptawell plates were coated with 1 μg of biotinylated cfc1-cyc citrullinated peptide or cf0-cyc control peptide (diluted in PBS, 0.1% BSA) overnight at 4°C. Rat serum samples (diluted 1:50 in PBS containing 0.05% Tween and 1% BSA) were added in duplicate and incubated for 1 hour in a 37°C humid chamber. Plates were washed before the addition of HRP-conjugated rabbit anti-rat IgG (Sigma P216; diluted 1:1,000 in PBS containing 0.05% Tween and 1% BSA), followed by incubation for 1 hour in a humid chamber at 37°C. Bound antibodies were revealed by the addition of 3,3',5,5'-tetramethylbenzidine (Sigma). The enzymic reaction was stopped with 0.5 M H_2_SO_4 _after 30 min, and absorbance was determined at 450 nm.

### *In vitro *T cell proliferation in response to stimulation with RSA and Cit-RSA

Inguinal lymph nodes from Lew.1AV1 rats immunised with RSA and Cit-RSA were removed 10 days after immunisation; single-cell suspensions were prepared and resuspended in Dulbecco's modified Eagle's medium supplemented with glutamine, penicillin, streptomycin, HEPES and 10% fetal calf serum (Life Technologies, Paisley, Renfrewshire, UK). Cells were cultured *in vitro *for 72 hours at 10^6 ^cells per well in triplicate (96-well flat-bottomed culture plates; Nunc) in the presence of RSA (10 μg/ml), Cit-RSA (10 μg/ml), PBS or concanavalin A (2 μg/ml; Sigma-Aldrich). [^3^H]Thymidine (PerkinElmer Life Sciences Inc., Boston, MA, USA), was added (1 μCi per well) for the final 16 hours of culture. Cells were harvested with a Tomtec cell harvester and [^3^H]thymidine incorporation was measured as counts per minute (c.p.m.) in a Wallac Trillux 1450 microbeta counter. Stimulation was calculated and expressed as stimulation index (C.p.m. after stimulation/C.p.m. of background).

### Antibodies

Recombinant anti-citrullinated protein single-chain variable fragment (scFv) antibody RA3, selected from RA-patient-derived phage display libraries and control scFv human anti-U1-70K have been described elsewhere [39]. Rabbit antibodies directed against chemically modified citrulline (anti-MC antibodies), developed by Dr Tatsuo Senshu and colleagues [5,41], were purchased from Upstate Biochemicals (Lake Placid, NY, USA). Polyclonal antibodies recognising PAD4 (SN823) were produced by immunising rabbits with PAD4 isotype-specific peptides (amino acids 210 to 225 and 517 to 531) and by affinity purification with a CNBr-activated Sepharose 4B column, as described previously [10]. Preimmune rabbit IgG was used as control for PAD4.

### Immunohistochemical analysis

Zamboni-fixed [42] cryopreserved sections of synovial tissue from CII-immunised DA rats were stained for expression of citrullinated proteins and PAD4. Endogenous peroxidase activity was blocked by treatment for 30 min in darkness at room temperature with 1% hydrogen peroxide and 2% sodium nitride dissolved in PBS. After washing with PBS, 2% normal goat serum was added to block non-specific binding sites. Sections were then incubated with 200 μl of primary antibody (RA3 (undiluted) or anti-PAD4 (diluted 1:24)) overnight in a humid chamber at 4°C. An isotype-matched recombinant human anti-U1-70K antibody and a preimmune rabbit IgG were used as respective controls. Slides were then washed in PBS before incubation with 200 μl of appropriate biotin-labelled secondary antibody (donkey anti-human IgG (Jackson Immuno Research) diluted 1:1,000 or goat anti-rabbit IgG (Vector, Burlingame, CA, USA) diluted 1:800) for 1 hour at room temperature. After further washing, 200 μl of avidin-biotin-HRP (Vectastain; Vector) prepared in accordance with the manufacturer's directions was applied for 60 min at room temperature. A final wash was followed by addition of the substrate diaminobenzidine (Peroxidase Substrate Kit; Vector) for 5 min. The colour reaction was stopped by washes in tap water, after which sections were counterstained with Mayer's haematoxylin. Finally the slides were dried and mounted with buffered glycerol, and evaluation was performed by microscopy with a Polyvar II microscope (Reichert-Jung, Vienna, Austria) connected to a charge-coupled device colour camera (DXC-750P; Sony Corp., Tokyo, Japan).

In addition, anti-MC antibodies were used to confirm the citrulline staining obtained with RA3. Before incubation with primary antibody, sections were treated for 3 hours at 37°C in a chemical modification solution consisting of one part solution A (0.025% (w/v) FeCl_3_, 4.6 M H_2_SO_4_, 3.0 M H_3_PO_4_) plus one part solution B (0.5% diacetyl monoxime, 0.25% antipyrine, 0.5 M acetic acid) (anti-Citrulline (Modified) Detection Kit; Upstate Biochemicals). Control sections were incubated in a solution containing one part solution A and one part Milli-Q water. After extensive washing in PBS, slides were incubated for 40 min at room temperature with 1% H_2_O_2_, washed in PBS and incubated for 30 min at room temperature with 5% normal goat serum in PBS plus 1% BSA. Primary antibody (anti-MC), diluted 1:1,000 in PBS plus 1% BSA, was added and slides were incubated overnight at room temperature. Slides were then washed in PBS, followed by incubation for 30 min at room temperature with secondary antibody (biotinylated goat anti-rabbit IgG; Vector) diluted 1:800 in PBS plus 1% BSA. The subsequent steps were performed as described above.

### Statistical analysis

All data were evaluated with the Mann–Whitney *U*-test for independent groups, except for arthritis incidence, which was evaluated by Kaplan–Meier survival analysis.

## Results

### Presence of citrullinated proteins and PAD4 in the arthritic joints correlates with the degree of inflammation

**Figure 2:**
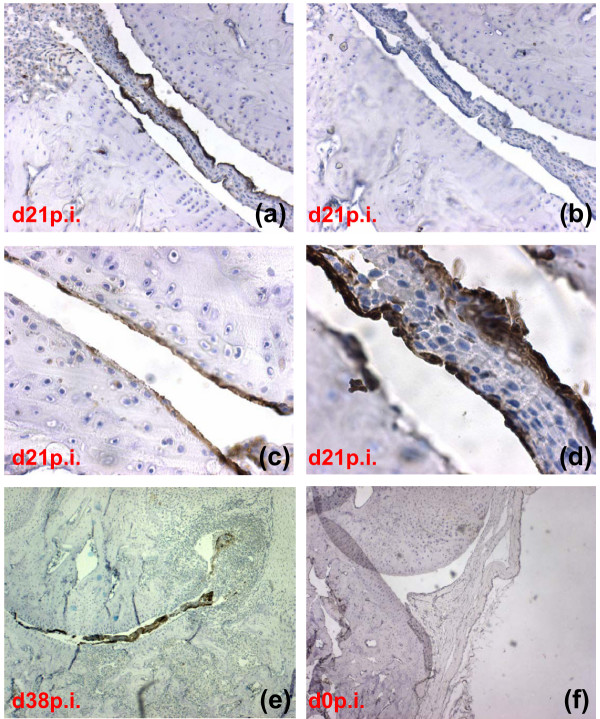
Citrullinated proteins are present in the arthritic joint. Positive citrulline staining was found in extracellular deposits **(a,e)**, cartilage **(c) **and infiltrating cells **(d)**. Unimmunised animals were negative for citrulline **(f)**. Immunohistochemical staining was performed with RA3, a human recombinant anti-citrullinated protein antibody. Control staining was performed with an isotyped-matched recombinant human anti-U1-70K antibody **(b)**. (Original magnifications: ×100 (a,b); ×250 (c); ×400 (d); ×40 (e,f)).

To examine the presence of citrullinated proteins and the enzyme PAD4 in the joints at different stages of experimental arthritis, histological analyses of rat ankle joints were performed by immunohistochemistry. Citrullinated proteins could be detected in the joints of arthritic animals with the RA3 antibody (Fig. 2 and Table 1). The first appearance of protein citrullination was noted after disease onset, at day 21 after immunisation, and increased staining was observed as the disease progressed into a more severe, chronic state (namely 28 and 38 days after immunisation). Unimmunised animals and time points before clinical signs of arthritis (namely 0, 3, 6 and 10 days after immunisation) as well as the time of disease onset (namely 15 days after immunisation) were negative for citrulline. Some infiltrating cells as well as the cartilage surface stained positively for citrulline and the major sources of citrullinated proteins in the arthritic joint were extracellular deposits, presumably fibrin deposits. The occurrence of citrullinated proteins in the joints is specific, because other investigated organs such as lung, ear, spinal cord, spleen, lymph nodes and salivary glands were all negative (data not shown).

For comparison with previous studies in humans [43] and mice [13] we also used antibodies targeting chemically modified citrulline. This method circumvents the risk of epitope blocking by residues flanking citrulline, because the citrulline side-chain becomes so bulky through the chemical modification that antibody recognition cannot be influenced by other amino acids [5,41]. Here we confirmed the results obtained with RA3. A similar staining pattern was observed, with positive cells, cartilage and extracellular deposits, whereas control stainings of non-modified sections were negative (data not shown).

**Figure 3:**
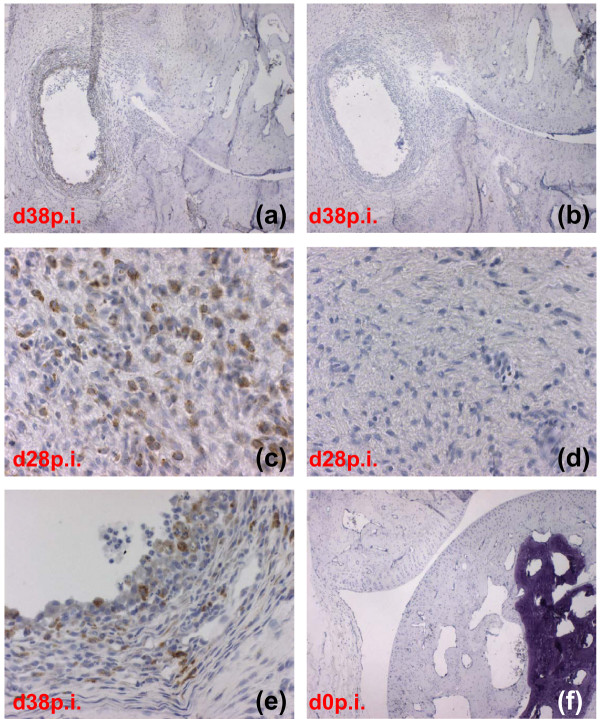
Peptidyl arginine deiminase 4 (PAD4) is present in the arthritic joint. PAD4 was detected in infiltrating cells **(a)**, localised to the cytoplasm of mononuclear cells **(c,e)**. Unimmunised animals were negative for PAD4 staining **(f)**. Immunohistochemical stainings were performed with a rabbit anti-PAD4 antibody. Control staining was performed with preimmune rabbit sera **(b,d) **(Original magnifications: ×40 (a,b,f); ×200 (c-e)).

PAD4, which has been reported to be present in mouse and human arthritic synovia [10,13], could also be detected in synovial tissue of this rat arthritis model from 21 days after immunisation (Fig. 3 and Table 1). The number of positive cells localised to the synovial infiltrate increased by days 28 and 38 after immunisation. PAD4 was not evident in healthy synovial tissue, nor in sections from 3, 6 and 10 days after immunisation; although an apparent inflammation was observed, PAD4 could not be detected 15 days after immunisation.

### Citrullination of a non-immunogenic antigen breaks B cell tolerance

**Figure 4:**
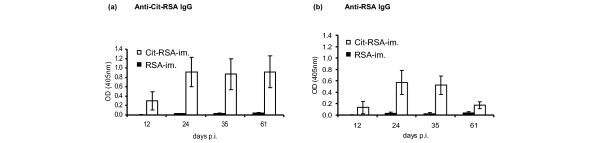
Immunisation with citrullinated rat serum albumin (Cit-RSA) breaks immunological tolerance on the B cell side. Immunisation with Cit-RSA (white bars) induced an antibody response against Cit-RSA **(a)**, cross-reacting with unmodified rat serum albumin (RSA) **(b) **at all time points investigated, whereas immunisation with RSA (black bars) did not. Sera were collected 12, 24, 35 and 61 days after immunisation, and total IgG was measured by ELISA as OD at 405 nm. Results are means ± SD (*n *= 7 animals per group), representative of two replicate experiments. **P < 0.01 at all time points.

Lew.1AV1 rats were immunised with the non-immunogenic autoantigen RSA or the modified counterpart Cit-RSA and the differences in induced immune responses were analysed *in vitro*. The kinetics of the antigen-specific IgG response was investigated and the results revealed not only a response towards the modified protein, but also cross-reactivity to the unmodified form of RSA (Fig. 4). Antibodies were detected from 12 days after immunisation, had increased by day 24 after immunisation and persisted for a further 40 days. Cross-reactivity was demonstrated at all time points. No B cell response was noted in animals immunised with unmodified RSA.

Animals immunised with Cit-RSA and RSA were also tested for anti-PAD IgG responses. The anti-PAD ELISA was negative both for animals immunised with Cit-RSA and for animals immunised with RSA (data not shown), indicating that the amount of PAD contaminating the Cit-RSA sample did not result in any false positive result in the Cit-RSA ELISA, described above.

In addition, by performing both an anti-CCP ELISA and a control ELISA containing cyclic arginine-containing peptides, we could confirm that the animals immunised with Cit-RSA produced antibodies against citrullinated epitopes and also antibodies recognising arginine epitopes (data not shown).

**Figure 5:**
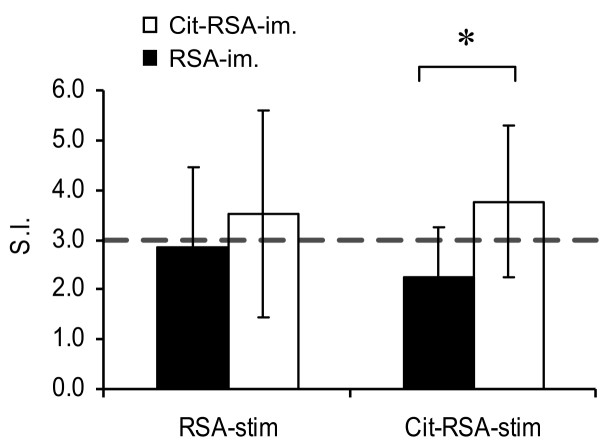
*In vitro *proliferative responses to rat serum albumin (RSA) and to citrullinated RSA (Cit-RSA). Stronger T cell responses to Cit-RSA (statistically significant) and to RSA (a trend) were observed in animals immunised with Cit-RSA (white bars) than in animals immunised with unmodified RSA (black bars). As previously shown (Fig. 4), the animals immunised with Cit-RSA developed a B cell response to RSA and to Cit-RSA, whereas the animals immunised with RSA did not. The dotted line suggests a hypothetical threshold that it is necessary to reach to induce B cell help and subsequent antibody production. Single cell suspensions were prepared from inguinal lymph nodes 10 days after immunisation, and stimulation index (S.I.) was calculated after 72 hours of culture in the presence of RSA or Cit-RSA (10 μg/ml). Results are means ± SD (*n *= 7 animals per group), representative of two replicate experiments. *P < 0.05.

The T cell response was evaluated by [^3^H]thymidine incorporation 10 days after immunisation, as depicted in Fig. 5. Here, a proliferative response was demonstrated in animals immunised with unmodified RSA, both when stimulated with the same antigen and when stimulated with the citrullinated antigen. Cells from animals immunised with Cit-RSA showed a stronger proliferation than the RSA-immunised rats in response to the modified protein. The same tendency was observed when using RSA as a stimulus, although it was not statistically significant.

### Immunisation with Cit-CII increases arthritis incidence and accelerates clinical onset of disease

**Figure 6:**
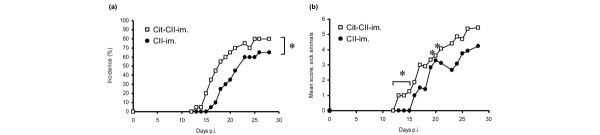
Citrullination of collagen type II (CII) increases its arthritogenic properties. Lew.1AV1 rats developed disease with higher incidence **(a) **and earlier onset as well as a trend towards higher mean arthritic score among the affected animals **(b) **when immunised with citrullinated CII (Cit-CII) (open squares) than when immunised with unmodified CII (filled circles). Rats were immunised with Cit-CII or unmodified CII and monitored for signs of arthritis for the next 30 days. Incidence (a) and mean score of sick animals (b) were calculated. Data include 20 animals per group from two pooled experiments. *P < 0.05.

Unmodified or citrullinated rat CII was, together with adjuvant, injected into Lew.1AV1 rats; arthritis development was monitored by blinded macroscopic evaluation. The citrullinated form of CII induced arthritis with significantly higher incidence and earlier onset than did the same amount of unmodified CII (Fig. 6). The incidence was 35% higher in the Cit-RSA group during the early phase of the disease and 15% higher during the end stage. Disease onset in this group occurred 13 days after immunisation, compared with 16 days after immunisation in the control group. A trend towards higher mean arthritis score was also observed in the affected animals in the Cit-CII group compared with the CII group, although this was not statistically significant.

## Discussion

The production of anti-citrullinated protein antibodies is almost 100% specific for patients with RA, indicating an important role for citrullinated proteins in the pathogenesis of RA. We therefore considered it of interest to investigate the ability to break immunological tolerance by the citrullination of endogenous proteins. Our data reveal that citrullination of the non-immunogenic antigen RSA induced an antibody response against the citrullinated protein with cross-reactivity to the unmodified protein at all time points investigated. The observation that anti-RSA IgG titres declined by 61 days after immunisation, as opposed to anti-cit-RSA IgG titres, might be due to affinity maturation of the B cell response with bias towards the citrullinated 'neoepitope'. Another possibility could be the formation of RSA antibodies and RSA immune complexes, which would increase their clearance from the circulation.

We could record an *ex vivo *proliferative T cell response to RSA as well as to Cit-RSA in rats immunised with modified protein and also in rats immunised with the unmodified protein, as demonstrated by stimulation index values over 1.0. When comparing the proliferative responses to Cit-RSA, cells from animals immunised with the citrullinated protein demonstrated a significantly stronger response than cells from animals immunised with the unmodified protein (*P *< 0.05). A similar trend was detected when comparing the responses of T cells to RSA, but the difference did not reach statistical significance. This indicates that RSA, together with a strong adjuvant, can induce an autoreactive T cell response, although this is not sufficient to induce B cell help. In contrast, immunisation with Cit-RSA induced a stronger T cell response that could confer B cell help, as depicted in Fig. 4.

Furthermore, Cit-CII induced arthritis with earlier onset and higher incidence than unmodified CII after immunisation. Considering the low arginine content of CII, our results indicate that protein modification by the conversion of arginine to citrulline is a highly potent mechanism for increased autoreactivity. Previous studies have demonstrated that anti-CII antibody titres do not reflect disease severity in this model of CIA and that stimulation *in vitro *with homologous CII does not induce T cell proliferation. We therefore chose not to analyse the humoral or cellular responses to CII in these animals.

Several post-translational protein modifications, especially those related to apoptosis [44,45], are associated with autoimmunity [46-49]. Inefficient clearance of these modified proteins in an inflammatory environment, conveying 'danger signals' to the immune system, might in combination with the appropriate MHC haplotype override tolerance mechanisms and activate autoreactive T cells. Citrullination interferes with organised protein structure, contributes to protein unfolding and to increased susceptibility to digesting enzymes [50,51], which could lead to altered antigen uptake, processing and presentation. Indeed, citrullinated peptides have been reported to bind more efficiently to the RA-associated HLA-DRB1*0401 haplotype than do non-citrullinated peptides [52]. As tolerance has not been established to such peripherally modified self-peptides, citrullination might increase the risk of activating pathogenic T cells. Our data therefore demonstrate not only the potency of citrullinated proteins to break tolerance, as evident from our experiments with RSA, but also the increased arthritogenic properties of Cit-CII.

Because anti-citrullinated protein antibodies can be detected in RA sera before clinical signs of disease [24], we speculate that citrullination of synovial proteins might occur at time points before RA becomes manifest. It is not ethically possible to examine the synovial tissue of healthy individuals at risk of developing RA. However, this becomes feasible with the use of an experimental model. In the present study we examined, for the first time, the kinetics of the presence of citrullinated proteins and the citrullinating enzyme PAD4 in joints during arthritis development. In this experimental model of homologous CIA with 100% disease incidence, citrullinated proteins and PAD4 were not detectable before clinical signs of arthritis. Rather, as inflammation proceeded, increasing amounts of citrullinated proteins and PAD4 were detected specifically in the joints. Extracellular deposits constitute the major source of citrullinated proteins in the inflamed joint, although both cells and cartilage also contribute to the positive staining. Although PAD4 has been described to have a nuclear localisation [12], our stainings clearly demonstrate the presence of this enzyme in the cytoplasm of infiltrating mononuclear cells. Our inability to detect nuclear staining of PAD4 might be due to the absence of permeabilising agents during staining procedures. Our finding that citrullinated synovial proteins could not be encountered before disease onset might explain the failure in detecting anti-citrullinated protein antibodies in animal models. Perhaps the presence of citrullinated proteins in these arthritis models is not disease-initiating, but rather a result of inflammation. This is also supported by the observation that citrullinated proteins can be detected in inflamed synovial tissue of non-RA patients lacking the anti-citrulline-specific B cell response [53,54].

Taking together our findings and those of others, we hypothesise that in individuals with the appropriate genetic background a subclinical inflammation that induces citrullination of endogenous proteins will generate a pathogenic immune response against the modified proteins and that through the production of autoantibodies a chronic inflammation will be established. An immune response towards any citrullinated protein is clearly not enough to induce clinical arthritis, as demonstrated by the lack of signs of disease in the Cit-RSA-immunised animals. Both genetic and environmental factors most probably contribute to the development of arthritis. The initiation of citrulline reactivity does not necessarily need to occur in the joints. For example, smoking has been reported to be an environmental risk factor for RA in individuals with MHC shared epitope [55]. It is plausible that smoking could induce such an inflammatory reaction in lungs with the formation of citrullinated proteins. Why an autoreactivity to citrullinated proteins would precipitate in arthritis and not in other organ-specific inflammatory diseases is currently unknown. However, several reports indicate that joints are especially sensitive to inflammatory stimuli. The experimental model oil-induced arthritis demonstrates that administration of the non-antigenic adjuvant mineral oil at a distant location (namely the tail base) induces arthritis and no other disease in rats [56]. Additionally, systemic overexpression of proinflammatory cytokine TNF generates a joint-specific inflammation [57]. Similarly, interleukin-1Ra knockout mice develop arthritis [58].

## Conclusion

Our data reveal the potency of citrullination to break tolerance against the ubiquitous systemic self antigen RSA and to increase the arthritogenicity of the tissue-specific protein CII. Furthermore, we have demonstrated that the amounts of citrullinated proteins and the enzyme PAD4 in the arthritic joints of experimental animals correlated with the severity of inflammation, while no citrullinated proteins or PAD4 could be detected in healthy joints. On the basis of our new data and previous findings on this topic we hypothesise that a subclinical inflammation might induce citrullination of endogenous proteins. This citrullination does not necessarily need to occur in joints but can occur elsewhere in the body. In combination with a certain genetic context, anti-citrullinated protein antibodies will be generated, which will potentiate an inflammatory trigger in joints and thereby initiate RA.

## Abbreviations

Anti-CCP = anti-cyclic citrullinated peptide; anti-MC antibodies = antibodies against chemically modified citrulline; BSA = bovine serum albumin; CIA = collagen-induced arthritis; CII = collagen type II; Cit-CII = citrullinated CII; Cit-RSA = citrullinated RSA; DA = Dark Agouti; ELISA = enzyme-linked immunosorbent assay; FIA = Freund's incomplete adjuvant; HRP = horseradish peroxidase; MHC = major histocompatibility complex; PAD = peptidyl arginine deiminase; PBS = phosphate-buffered saline; RA = rheumatoid arthritis; RSA = rat serum albumin.

## Competing interests

The author(s) declare that they have no competing interests.

## Authors' contributions

KL was responsible for most of the experiments and data analysis as well as drafting the manuscript. HEH, together with KL, was responsible for study design coordination and compilation of the manuscript. SN and ERV performed the citrullination of proteins and the production of antibodies (RA3, anti-U1-70K, anti-PAD4 and preimmune rabbit sera). KP performed the immunisation, scoring and sectioning of rat ankle joints of the DA rats. WJV, LK and AJWZ contributed to interpretation and discussion of data. All authors read and approved the final manuscript.

## Figures and Tables

**Table 1:** Correlation of presence of citrullinated proteins and peptidyl arginine deiminase 4 with degree of inflammation

Days after immunisation	Arthritis score	Cell infiltration	Cartilage destruction	Citrulline-positive deposits	Citrulline-positive cells	Citrulline-positive cartilage	PAD4-positive cells
0	0.0	0.0	0.0	0.0	0.0	0.0	0.0
3	0.0	0.0	0.0	0.0	0.0	0.0	0.0
6	0.0	0.0	0.0	0.0	0.0	0.0	0.0
10	0.0	0.0	0.0	0.0	0.0	0.0	0.0
15	3.3 ± 2.4	1.3 ± 0.5	0.0	0.0	0.0	0.0	0.0
21	9.8 ± 3.3	1.8 ± 0.5	0.5 ± 0.6	1.0	0.5 ± 0.6	0.8 ± 1.0	1.0 ± 0.5
28	11.0 ± 3.5	3.0	2.5 ± 0.6	1.8 ± 1.0	1.3 ± 0.5	1.5 ± 1.3	1.5 ± 0.4
38	11.8 ± 2.6	3.0	3.0	2.7 ± 0.6	1.8 ± 0.5	0.5 ± 0.6	2.2 ± 0.5

PAD4, peptidyl arginine deiminase 4. Arthritic score, cell infiltration, cartilage destruction and presence of citrullinated proteins in extracellular deposits, cells and cartilage were evaluated at different time points of collagen-induced arthritis. All data are from four rats, evaluated in a blinded manner by two independent evaluators and are means ± SD. Arthritis score ranges from 0 to 16 as explained in the Materials and methods section. Arbitrary units were used for the other features, ranging from 0, indicating no cell infiltration, cartilage destruction, presence of citrullinated proteins or PAD4, to 3, indicating extensive cell infiltration, cartilage destruction, presence of citrullinated proteins and PAD4.
